# Exosome‐transmitted podoplanin promotes tumor‐associated macrophage‐mediated immune tolerance in glioblastoma

**DOI:** 10.1111/cns.14643

**Published:** 2024-03-12

**Authors:** Mengwan Wu, Ying Shi, Yuyang Liu, Hongxiang Huang, Jiajia Che, Jing Shi, Chuan Xu

**Affiliations:** ^1^ Department of Oncology, Sichuan Academy of Medical Sciences, Sichuan Provincial People's Hospital, School of Medicine University of Electronic Science and Technology of China Chengdu Sichuan China; ^2^ Yu‐Yue Pathology Scientific Research Center Chongqing China; ^3^ Jinfeng Laboratory Chongqing China; ^4^ Department of Neurosurgery 920th Hospital of Joint Logistics Support Force Kunming China; ^5^ Department of Oncology, The First Affiliated Hospital Nanchang University Nanchang China

**Keywords:** exosome, GBM, glioma, immunosuppressive TME, macrophage, PDPN

## Abstract

**Aims:**

Glioblastoma is the most frequent and aggressive primary brain tumor, characterized by rapid disease course and poor treatment responsiveness. The abundance of immunosuppressive macrophages in glioblastoma challenges the efficacy of novel immunotherapy.

**Methods:**

Bulk RNA‐seq and single‐cell RNA‐seq of glioma patients from public databases were comprehensively analyzed to illustrate macrophage infiltration patterns and molecular characteristics of podoplanin (PDPN). Multiplexed fluorescence immunohistochemistry staining of PDPN, GFAP, CD68, and CD163 were performed in glioma tissue microarray. The impact of PDPN on macrophage immunosuppressive polarization was investigated using a co‐culture system. Bone marrow‐derived macrophages (BMDMs) and OT‐II T cells isolated from BALB/c and OT‐II mice respectively were co‐cultured to determine T‐cell adherence. Pathway alterations were probed through RNA sequencing and western blot analyses.

**Results:**

Our findings demonstrated that PDPN is notably correlated with the expression of CD68 and CD163 in glioma tissues. Additionally, macrophages phagocytosing PDPN‐containing EVs (EVs^PDPN^) from GBM cells presented increased CD163 expression and augmented secretion of immunoregulatory cytokine (IL‐6, IL‐10, TNF‐α, and TGF‐β1). PDPN within EVs was also associated with enhanced phagocytic activity and reduced MHC II expression in macrophages, compromising CD4^+^ T‐cell activation.

**Conclusions:**

This investigation underscores that EVs^PDPN^ derived from glioblastoma cells contributes to M2 macrophage‐mediated immunosuppression and is a potential prognostic marker and therapeutic target in glioblastoma.

## Graphical abstract

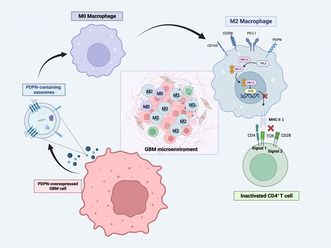

## 1 INTRODUCTION

Glioblastoma multiforme (GBM), accounting for 49.1% of all malignant central nervous system tumors, is characterized by a high degree of malignancy and rapid progression. Despite the application of multimodal standard therapies encompassing surgery, chemotherapy, and radiotherapy, the median survival duration remains a dismal 8 months.^1^ Given the limited efficacy of existing treatment modalities and the resulting poor median survival, the pursuit of novel therapeutic strategies for glioblastoma is of paramount importance.

In recent decades, high‐throughput technologies have allowed for extensive evaluation of tumor immune microenvironment. Through different immunotherapeutic strategies—either preventing immune escape or restoring anti‐tumor immunity, immune therapy, especially the immune checkpoint blockade, has demonstrated considerable efficacy in improving survival outcomes across various extracranial tumors.^2^, ^3^ However, success has not been replicated in the context of glioblastoma,^4^, ^5^ which was attributed to the “cold” immune microenvironment typified by non‐responsive and dysfunctional cytotoxic immune cells. The inherent molecular heterogeneity and the unique TME of the brain, as well as growth factors and chemoattractants secreted by GBM cells, exert a profound influence on immune cell trafficking. Therefore, it is imperative to elucidate the relationships between molecular heterogeneity and immune phenotypes at both intertumoral and intratumoral levels to inform potential therapeutic regimens.

Within the immune microenvironment of glioblastoma, a multitude of immune cells partake in shaping the disease pathology, among which macrophages play a particularly pivotal role. These cells comprise up to 30%–50% of cellular components in glioma tissues.^6^ Typically, unpolarized macrophages (M0) could be activated into pro‐inflammatory M1 or immunosuppressive M2 types, a process crucial to the host immune response.^7^, ^8^ Research has suggested that complex crosstalk has been established between glioma and glioma‐associated macrophages (GAMs).^9^ Extracellular vehicles (EVs), which act as messengers for substance transfer, play a significant role in influencing the function of GAMs. Mounting evidence has shown that exosomes with a diameter range of 30–150 nm containing a variety of bioactive compounds such as proteins, nuclear acids, and lipids convey signals in the interaction between glioma cells and GAMs.^10^, ^11^ In this circumstance, there is a tendency for GAMs to preferentially differentiate into the M2 subtype, which correlates with suppressed T‐cell function.^12^

This suppression of T‐cell function by GAMs poses a challenge. Yet, macrophages possess intrinsic capabilities that can be harnessed therapeutically: they bear both classes I and II major histocompatibility complexes (MHC), which are responsible for identifying and presenting foreign antigens into T cells. Particularly, MHCII‐restricted antigen presentation is a key mechanism to directly maintain functional cytotoxic T‐cell responses within brain tumors.^13^ Therefore, elevating MHC II expression is supposed to be a promising strategy to restore GAMs and T‐cell function.

Considering that surface molecules and cytokines have a prominent role in microglia/macrophage‐glioma cell interactions, we explored the potential factor included in regulating the function and infiltration of GAMs. Podoplanin (PDPN) is a mucin‐like transmembrane glycoprotein that plays diverse roles in the regulation of lymphangiogenesis, immune responses, thrombosis, and processes of tumorigenesis and metastasis.^14^, ^15^ Recent studies have revealed that PDPN is upregulated in various cancers, especially high in tumors derived from immune‐privileged organs, such as glioma and testis cancer.^16^, ^17^ This upregulation has been found to correlate with malignant phenotype, treatment resistance, and poor prognosis.^14^, ^15^ PDPN‐positive cancer‐associated fibroblasts have been implicated with immune suppression in pancreatic adenocarcinoma and lung cancer.^18^, ^19^ In gliomas, PDPN expression is elevated in accordance with tumor malignancy^20^ and correlated with radioresistance.^21^, ^22^ However, how PDPN regulates macrophage polarization in glioma remains unclear.

## 2 MATERIALS AND METHODS

### 2.1 Data collection

The Bulk RNA‐seq data of LGG and GBM samples were downloaded from The Cancer Genome Atlas (TCGA, https://xenabrowser.net/) and the China Glioma Genome Atlas (CGGA, http://www.cgga.org.cn/) datasets.^23^ GTEx brain RNA‐Seq data were downloaded from the GTEx Portal (https://gtexportal.org/home/). To avoid the effects of batch effect, the TCGA TARGET GTEx cohort from Xena browser (University of California) was used (https://xena.ucsc.edu/), which contained gene expression RNAseq data of glioma (TCGA) and healthy brain tissue (Genotype‐Tissue Expression, GTEx). For anatomic structural expression analyses, RNA‐seq data of GBM was collected from Ivy Glioblastoma Atlas Project (http://glioblastoma.alleninstitute.org/). To illustrate the immune microenvironment characteristics of human glioblastoma, the single‐cell sequencing data containing 201,986 cells from 18 human primary GBM samples were obtained from the Single Cell Portal platform (http://singl ecell.broadinstitute.org) (accession number SCP1985, GSE182109).^24^ For PDPN protein expression analyses, immunohistochemistry images were downloaded from Human Protein Atlas (HPA) website (https://www.proteinatlas.org/).^25^

### 2.2 Tumor‐infiltrating immune cells estimation

To identify the relationship between PDPN expression and the activity of immune cells, ssGSEA algorithm (Hallmark Gene sets from Molecular Signatures Database, MSigDB) was conducted with GSVA (1.34.0) in TCGA database. CIBERSORT algorithm was performed to predict the composition of infiltrating immune cells in samples from TCGA and CGGA.

### 2.3 Single‐cell sequencing analysis

The R package Seurat (5.0.1) was used to process scRNA‐seq data. Cells containing genes that can only be detected in fewer than three cells and cells with <200 detected genes were excluded from subsequent analysis. Percentage Feature Set function was conducted to calculate the mitochondria gene expression. High‐quality cells with <5% mitochondrial transcripts were filtered and retained. Filtered cells were clustered using FindNeighbors and FindClusters functions of Seurat. To identify marker genes of each cluster, FindAllMarkers function was used. Genes expressed in at least 25% of cells in an interest cluster were chosen and meanwhile filtered using an absolute log_2_ (fold change) of 0.25 and a *p*‐value of 0.01. Manual annotation was performed by combining use of SingleR package (2.4.0) and a comprehensive reference to Cellmarker (http://biocc.hrbmu.edu.cn/CellMarker/),^26^ PanglaoDB (https://panglaodb.se/),^27^ and Enrichr (https://maayanlab.cloud/Enrichr/)^28^ databases.

### 2.4 Trajectory analysis

Single‐cell pseudotime analysis was performed by Monocle 2 (2.18.0).^29^ A set of ordering genes that were expressed in at least 10% of all cells was selected. “DDRTree” method was used to reduce the dimensionality.

### 2.5 Cell–cell interaction analyses

According to PDPN expression at single cell level shown on violin map (Figure S1), tumors were divided into PDPN high and low groups. To explore intercellular communication networks in tumors with high or low PDPN expression, cell–cell interaction analyses were conducted with CellChat package (1.1.3).^30^ The CellChatDB human database was used for analysis. The “identifyOverExpressedGenes” and “identifyOverExpressedInteractions” functions were applied to identify differential expression genes and pathways. The “netVisual” function was used for visualization.

### 2.6 Cell culture and transfection

Human GBM cell U87‐MG, mouse glioma cell GL261, and human monocyte leukemia cell line THP‐1 were used in this study. Cells utilized in our experiments were sourced from laboratory storage. Short tandem repeat (STR) testing was performed to confirm cell identity. U87‐MG and GL261 were cultured in maintained in DMEM medium (Gibco, USA) with 10% fetal bovine serum (FBS) (Gibco, USA), and THP‐1 was maintained in RPMI 1640 medium (Gibco, USA) supplemented with 10% FBS in a humidified chamber containing 5% CO_2_ at 37°C. For differentiation into macrophages, THP‐1 was treated with 100 ng/mL phorbol 12‐myristate 13‐acetate (PMA, tsbiochem, China) in RPMI 1640 medium for 48 h. PDPN‐overexpressed U87‐MG and GL261 cell lines were established using lentivirus containing pLVX‐PDPN‐Puro and pLVX‐Puro (Vector) purchased from Exongen (Exongen, China).

### 2.7 Multiplexed fluorescence immunohistochemistry (MF‐IHC)

Glioma tissue microarray was purchased from Shanghai SuperChip Biotech Co. Ltd. A five‐color Multiplexed Fluorescence Immunohistochemical Staining Kit (Absin, mIHC‐9963‐5‐EA) was used for MF‐IHC.^31^ The following primary antibodies were used for the incubation: GFAP (1:200, CST, #80788), PDPN (1:4000, Proteintech, #67432‐1‐Ig), CD68 (diluted 1:200, CST, #76437), and CD163 (1:500, CST, #93498). Nuclei were stained with DAPI after being labeled with human antigens. The Pannoramic Scanner (3D HISTECH, Hungary) was employed to obtain the MF‐IHC staining images. The HALO software (HALO, Indica labs) was used to quantify positive cells at the single‐cell level. The images were visualized and analyzed with Caseviewer (version 2.4) image analysis tools.

### 2.8 Coculture of U87‐MG and macrophages

In the coculture model,^32^ 24‐well plates containing polycarbonate transwell inserts with 0.4‐μm pores (Corning) were used. GBM cell U87‐MG (5 × 10^4^) were seeded in the insert, while THP‐1 derived M0 macrophages (5 × 10^4^) were seeded in the well‐bottom. GW4869 (Sigma‐Aldrich, 10 μM) was employed to block exosome secretion. After coculture for 48 h, macrophages were collected for the following analysis.

### 2.9 Purification and characterization of the extracellular vesicles

Before cell culture, FBS was centrifuged at 100,000 *g* overnight to isolate exosomes. Tumor cells were cultured in RPMI 1640 medium supplemented with 10% exosome‐depleted FBS. Differential centrifugation purification was used to isolate extracellular vesicles from cell culture supernatants after 72 h cell cultures.^33^ To get rid of dead cells and cell debris, culture supernatants were first centrifuged at 4000 *g* for 15 min at 4°C. After 45 min of centrifugation at 16,500 *g*, collected supernatants were ultracentrifuged for 2 h at 4°C at 110,000 *g* (Beckman Coulter, Optima XPN‐100). The pelleted exosomes were resuspended in sterile PBS, and the centrifugation at 110,000 *g* for another 90 min at 4°C. Nanoparticle Tracking Analysis (Particle Metrix, ZetaVIEW) and transmission electron microscopy (Hitachi, HT‐7700) were used to characterize extracellular vesicles. Western blot was used to verify the expression of EVs' marker proteins CD63 and CD81.

### 2.10 Immunofluorescence

Macrophages were seeded into 35‐mm confocal dishes (Corning, USA). After coculturing with PKH67‐labeled EVs for 1 h, macrophages were gently washed and fixed with 4% paraformaldehyde, followed by permeabilization with 0.1% Triton X‐100. Latrunculin A (Lat A, 30 μM) was used to block phagocytosis of EVs. Cell images were captured by immunofluorescence microscopy (Nikon A1R+N‐STORM 4.0, Japan).

### 2.11 Flow cytometry

To identify PDPN and CD163 expression of macrophages, the experiments were divided into four groups: M0, M0 + EVs^U87MG‐PDPN^, M0 + EVs^U87MG‐VEC^, and M0 + EVs^U87MG‐PDPN^ + Latrunculin A (Lat A, 30 μM). CD68^+^ was used to label human macrophages, and CD68^+^ CD163^+^ was used to label human M2 macrophages. Macrophages from all groups were processed into single‐cell suspensions and stained with antibodies for 30 min on ice. The following flow cytometry antibodies were used for the incubation: PDPN (BioLegend, #337003), CD68 (BD Bosciences, #564943), and CD163 (BioLegend, #333609). After washing with PBS, the expression of PDPN and CD163 was detected using flow cytometry (BD Cantoll, San Jose, CA).

### 2.12 ELISA

To investigate the secretion of M2‐related cytokines, macrophages were seeded into six‐well plates at a density of 8 × 10^5^ cells per well. 50 μg EVs derived from U87‐MG^PDPN^ and U87‐MG^VEC^ were treated to macrophages with or without Latrunculin A (Lat A, 30 μM). After incubation for 24 h, the supernatant was collected to detect the concentration of human IL‐6, IL‐10, TNF‐α, and TGF‐β1 using Human IL‐6 ELISA Kit (abclonal, China), Human IL‐10 ELISA Kit (abclonal, China), Human TNF‐alpha ELISA Kit (abclonal, China), and Human Transforming Growth Factor Beta 1 ELISA Kit (abclonal, China) according to the manufacturer's instructions.

### 2.13 Phagocytosis analysis

To explore the effect of PDPN‐containing EVs on macrophage phagocytosis, macrophages were seeded into six‐well plates at a density of 8 × 10^5^ cells per well. After coculturing with 50 μg EVs isolated from human GBM cell U87‐MG with or without Latrunculin A (Lat A, 30 μM) for 24 h, 1 mg/mL pHrodo Green *E. coli* BioParticles Conjugate (Invitrogen, USA) was added to the macrophages (8 × 10^5^ cells) and incubated for 2 h at 37°C according to the manufacturer's instructions. Then macrophages were washed in ice‐cold phosphate‐buffered saline with 1% bovine serum albumin. The uptake of pHrodo green was measured by flow cytometry and data was analyzed by FlowJo (Version 10.8.1).

### 2.14 RNA sequencing analysis of GBM cell lines

U87‐MG^PDPN^ and U87‐MG^VEC^ were cultured and harvested. The total RNA was extracted and sequenced using the Illumina HiSeq 2500 platform (San Diego, USA). Raw reads were converted into fragments per kilobase of exon model per million mapped reads (FPKM) values for further analysis. Differentially expressed genes (DEGs) were identified using the DESeq2 package in R screened with *p* < 0.01, |log_2_FC| ≥ 2. Heatmaps were plotted using GraphPad Prism 7 (GraphPad Software Version 9.5.1) based on log_2_(FPKM + 1) values. The gene‐set enrichment analysis (GSEA) was performed using GSEA software (version 4.3.2). KEGG pathway enrichment analysis was conducted using KEGG database.

### 2.15 Isolation of bone marrow‐derived macrophages (BMDMs) and OT‐II cells

BALB/c and OT‐II mice were housed under specific pathogen‐free conditions. For experiments, 8‐ to 12‐week‐old mice were used. All procedures were done in accordance with the Institutional Animal Care and Use Committee of the School of Medicine of UESTC. BMDMs were prepared as described.^34^ In brief, bone marrow isolated from femurs and tibias of BALB/c mice and treated with ACK lysis buffer (Beyotime, China). Then the cells were cultured in RPMI 1640 (Gibco, USA) supplemented with 10% FBS (Gibco, USA), 1% Penicillin–Streptomycin Solution (biosharp, China), and 50 ng/mL macrophage colony stimulating factor (M‐CSF) (abclonal, China) for 6 days to stimulate differentiation into BMDMs. OT‐II cells were isolated from the spleen of an OT‐II mouse using a CD4^+^ enrichment kit (Invitrogen, USA) according to the manufacturer's instructions.

### 2.16 Macrophage polarization

To induce M2‐like polarization, THP‐1 cells were treated with 100 ng/mL PMA (tsbiochem, China) for 48 h, followed by addition of IL‐4 (abclonal, China) and IL‐13 (abclonal, China) (20 ng/mL) for another 48 h. For BMDMs, the cells were stimulated with 20 ng/mL IL‐4 (abclonal, China) for 24 h. Cells were harvested and analyzed by flow cytometry or immunofluorescence.

### 2.17 BMDMs and OT‐II cell adherence

To evaluate the antigen presentation ability of EVs^PDPN^ treated macrophages, BMDMs‐derived M2 were cocultured with EVs from GL261^PDPN^ or GL261^VEC^ for 48 h. Then BMDMs were pulsed with ovalbumin (100 μg/ml, Sigma Aldrich, A5503‐1G) for another 24 h and then cocultured with OT‐II cells for 1 h.^35^, ^36^ To eliminate unbound T cells, samples were rinsed with PBS three times gently and then fixed in 2% paraformaldehyde for 15 min. CD11b and CD4 antibodies were used to label BMDMs and CD4^+^ T cells. After incubation at 4°C overnight, three times washes with PBS were performed. After fluorescent secondary antibody (Yeasen, China) was incubated for 30 min at room temperature (25°C), images were taken. BMDMs and OT‐II cell adherence were analyzed by mean fluorescence intensity ratio of T cell (CD4: red) and BMDMs‐derived M2 (CD11b: green).^37^

### 2.18 Western blot assay

Human GBM cell U87MG‐derived EVs and cell lysates from M2 macrophages were collected in RIPA lysis buffer (Beyotime, China). The protein concentrations of purified EVs and total protein extracted from M2 cells were assessed using the bicinchoninic acid (BCA) protein assay kit (Vazyme, China). The cell lysates (40 μg) and sEVs proteins (5 μg) were separated by sodium dodecyl sulfate‐polyacrylamide gel electrophoresis and then transferred to PVDF membranes (Merck Millipore, USA). Membranes were blocked and incubated with primary antibodies at 4°C overnight. β‐tubulin and GAPDH were used for loading controls. After incubation of horseradish peroxidase (HRP)‐conjugated anti‐mouse or anti‐rabbit secondary antibodies (SA00001‐1 and SA00001‐2; 1:5000; Proteintech, China) at room temperature (RT) for 1 h, blots were visualized using ECL Plus western blotting detection system.

### 2.19 Statistical analysis

Statistical analyses were performed using R software (version 4.2.1) and GraphPad Prism 9 (Version 9.5.1). Normality was tested with the Shapiro–Wilk normality test. According to the normality of data, Student's *t*‐test and Mann–Whitney test were used to compare the means of two groups. For multiple groups, the parametric one‐way analysis of variance was used for normally distributed variables, while nonparametric Kruskal–Wallis test for non‐normally groups. Tukey or Dunn's tests were applied for post‐hoc comparisons. The correlations between continuous variables were investigated using Spearman correlation analysis. Survival analysis was performed by the Kaplan–Meier method. The mean ± standard deviation was shown in the figures. Significance was determined as *p < 0.05, **p < 0.01, ***p < 0.001, and ****p < 0.0001.

## 3 RESULTS

### 3.1 PDPN expression is associated with macrophage M2‐like polarization in gliomas

**FIGURE 1:**
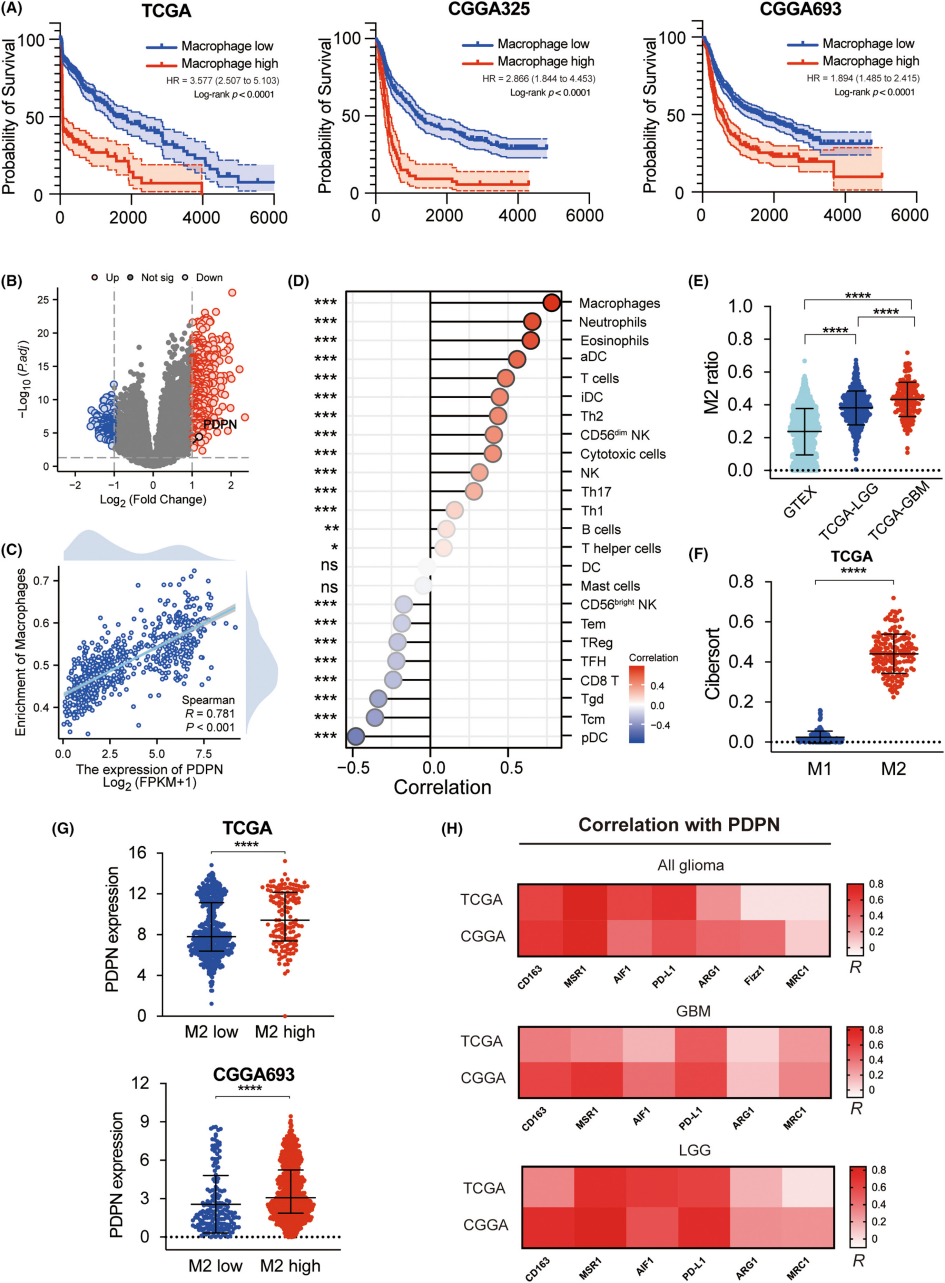
Correlation of PDPN expression and M2 macrophage infiltration in TCGA and CGGA. (A) Survival analyses were performed between macrophage high group and low group in TCGA and CGGA. (B) Volcano plot depicted DEGs of Macrophage high group and low group, screened with *p* < 0.05, |log_2_FC| ≥ 1. (C, D) Correlation analyses were performed between PDPN expression and infiltrating immune cells. (E) M2 ratio in normal brain tissues (GTEX), LGG (TCGA‐LGG), and GBM (TCGA‐GBM) were compared. (F) Infiltration of M1 macrophage and M2 macrophage of TCGA were presented based on cibersort algorithm. (G) PDPN expression in M2 high and low group in TCGA and CGGA693. (H) Correlation coefficient of PDPN and M2 marker in TCGA and CGGA, shown by heatmap. *p < 0.05, **p < 0.01, ***p < 0.001, ****p < 0.0001.

To Investigate the predominant immune constituents within the glioma TME, we probed the prognostic implications of macrophage presence utilizing data from TCGA, CGGA325, and CGGA693 databases. Immune infiltration analysis of TCGA dataset was performed with CIBERSORT algorithm. Patients were divided into macrophage high and low groups based on the cutoff values generated by xtile software. Our results, depicted in Figure 1A, reveal a marked disparity in overall survival (OS) between the two cohorts across all databases, with the macrophage‐dense group exhibiting reduced OS. To identify the latent key factors affecting macrophage infiltration, we explored the gene expression profile of 702 samples in the TCGA database and analyzed DEGs screened with log_2_FC ≥ 1, FDR < 0.05 of patients in two groups. 324 genes were found upregulated and 588 genes downregulated (Figure 1B). Notably, PDPN was significantly upregulated in patients of the macrophage high group. To figure out whether upregulated PDPN is associated with immunosuppressive macrophages in glioma, correlation analyses were conducted. As presented in Figure 1C,D, PDPN expression was positively correlated with macrophage infiltration compared with other immune cells. To determine macrophage subtypes composition in glioma, macrophage polarization states were investigated in GTEX, TCGA, and CGGA databases. The proportion of M2 macrophages was significantly increased in GBM than in LGG and normal brain tissues (Figure 1E). Consistently, M2 ratio was considerably higher than M1 in TCGA and CGGA (Figure 1F, Figure S2A,B). Further, PDPN expression was significantly greater in the M2‐dominant subgroup within these cohorts (Figure 1G, Figure S2C), showing a positive correlation with M2 markers (CD163, MSR1, AIF1, ARG1, MRC1) and PD‐L1 (Figure 1H). Bulk RNA‐seq data analysis indicated that PDPN was associated with macrophage M2‐like polarization in gliomas.

### 3.2 PDPN‐associated TME immunophenotype revealed by single‐cell sequencing

**FIGURE 2:**
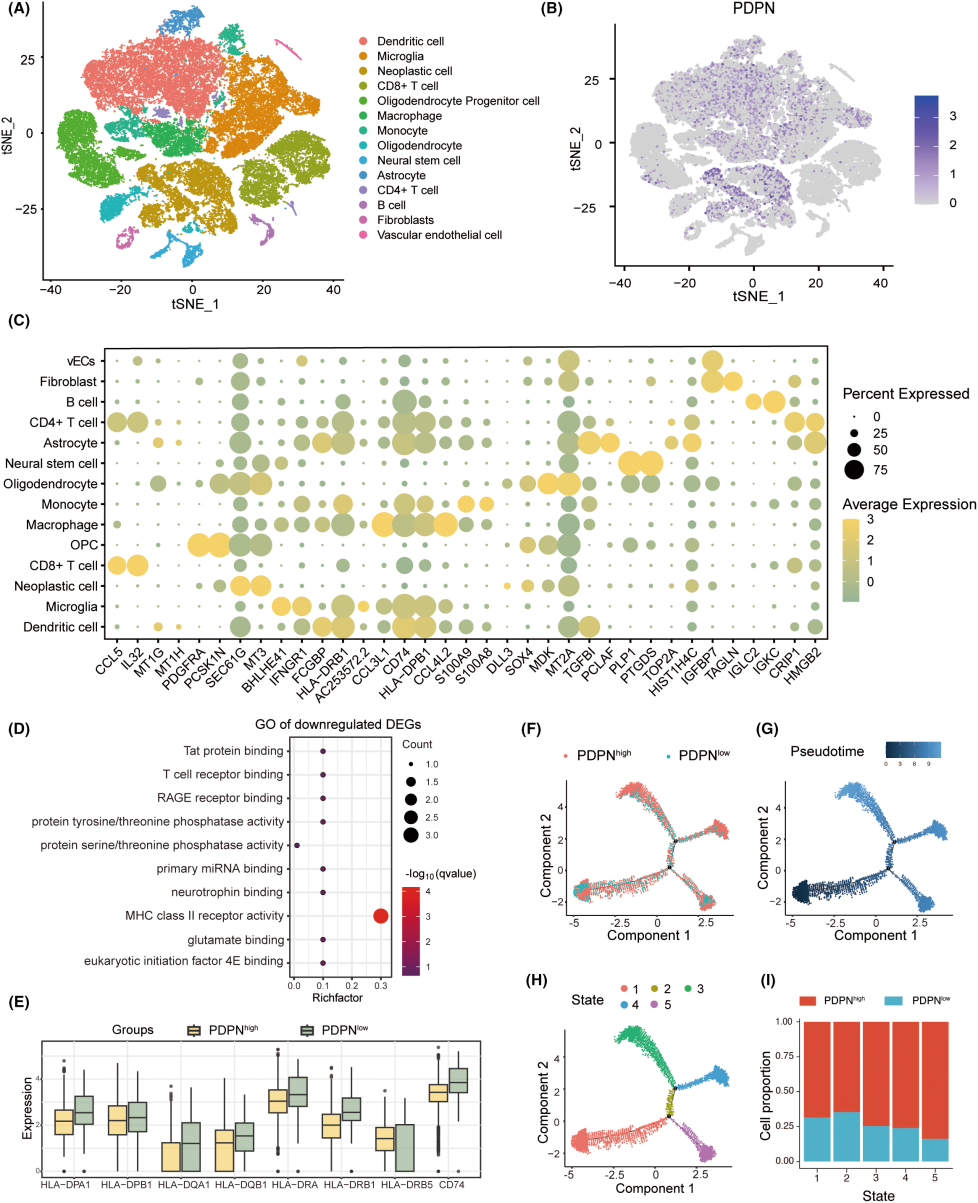
PDPN‐related immune cell profile at single cell level. (A) The 14 clusters were dimension‐reduced and visualized using t‐SNE. (B) PDPN expression of all 14 clusters was shown on the t‐SNE plot. (C) The top DEGs among all 14 clusters were depicted on dotplot. (D) GO enrichment analysis of downregulated genes in PDPN high group compared to the low group. (E) The expression of MHC class II molecules and CD74 in macrophages of PDPN high and low group. (F) Pseudotime trajectory analysis of neoplastic cells based on PDPN expression. (G, H) Trajectory plot based on pseudotime and state. (I) Cell portion of PDPN high and low in five states.

PDPN‐associated molecular characteristics of TME in 18 GBM samples were then investigated using single‐cell sequencing techniques. Combining the “singleR” package with manual annotation, cellular constituents were identified into 14 distinct clusters (Figure 2A). The distribution of PDPN across these clusters was visualized in Figure 2B and the DEGs characterizing the 14 cell types were shown in Figure 2C. According to GO enrichment analysis, downregulated genes were enriched in major histocompatibility complex (MHC) class II receptor activity and T‐cell receptor binding pathways and upregulated genes were enriched in the cytoskeleton regulation pathway (Figure 2D, Figure S3). Similarly, the expression of MHC class II molecules and the dedicated chaperone protein, CD74, decreased in macrophages of the PDPN high group (Figure 2E). To determine the sequential patterns of cellular changes, pseudotime trajectory analysis was conducted with monocle2 package. Five cell states were identified. Tumor cells would gradually evolve from state 1 to state 2–5. Interestingly, PDPN expression increased along the pseudotime and peaked in the last state (state 5) (Figure 2F–I).

### 3.3 PDPN‐related cellular interaction network

**FIGURE 3:**
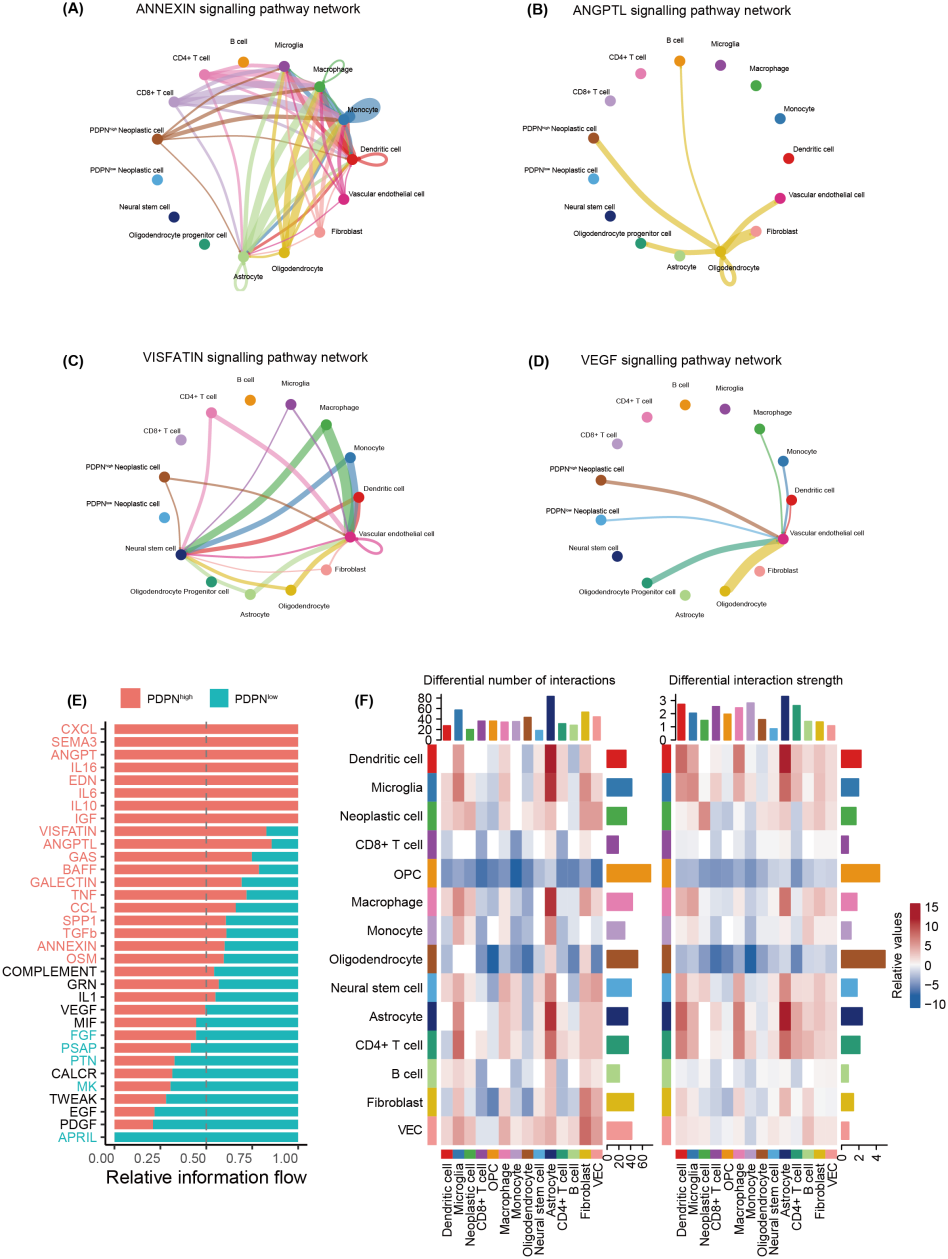
Cellular communication groups based on PDPN expression. (A–D) The cellular interaction in M2‐related pathways and VEGF pathway shown on circle plots. (E) Upregulated pathways enriched in PDPN high (red) and low groups (green). (F) The differential number of interactions and interaction strength after subtracting the value of the PDPN‐low group from the PDPN‐high group shown on heatmaps.

Compared with PDPN‐low neoplastic cells, how PDPN‐high neoplastic cells interact with other cells and influence the TME has been investigated with “Cellchat” package (Figure S4). M2 polarization‐associated signaling pathways were activated in PDPN‐high neoplastic cells, including ANNEXIN,^38^ ANGPTL,^39^ VISFATIN^40^ pathway (Figure 3A–C). Moreover, the VEGF pathway was also upregulated in PDPN‐high neoplastic cells (Figure 3D). Figure 3E consistently showed that the activity of IL6, IL10, TNF, and TGFb pathways was enhanced in the PDPN‐high group. The differential number of interactions and interaction strength after subtracting the value of the PDPN‐low group from the PDPN‐high group was displayed in Figure 3F. We can find that the number of interactions between neoplastic cells and macrophages was elevated while interaction strength was basically the same between the two groups. Intriguingly, the communication patterns between CD4^+^ T cells and other cellular populations exhibited a marginal increase in both frequency and strength, whereas those involving CD8^+^ T cells displayed a declining trend (Figure 3F).

### 3.4 Heterogeneous expression of PDPN at the intertumor and intratumor levels in gliomas

**FIGURE 4:**
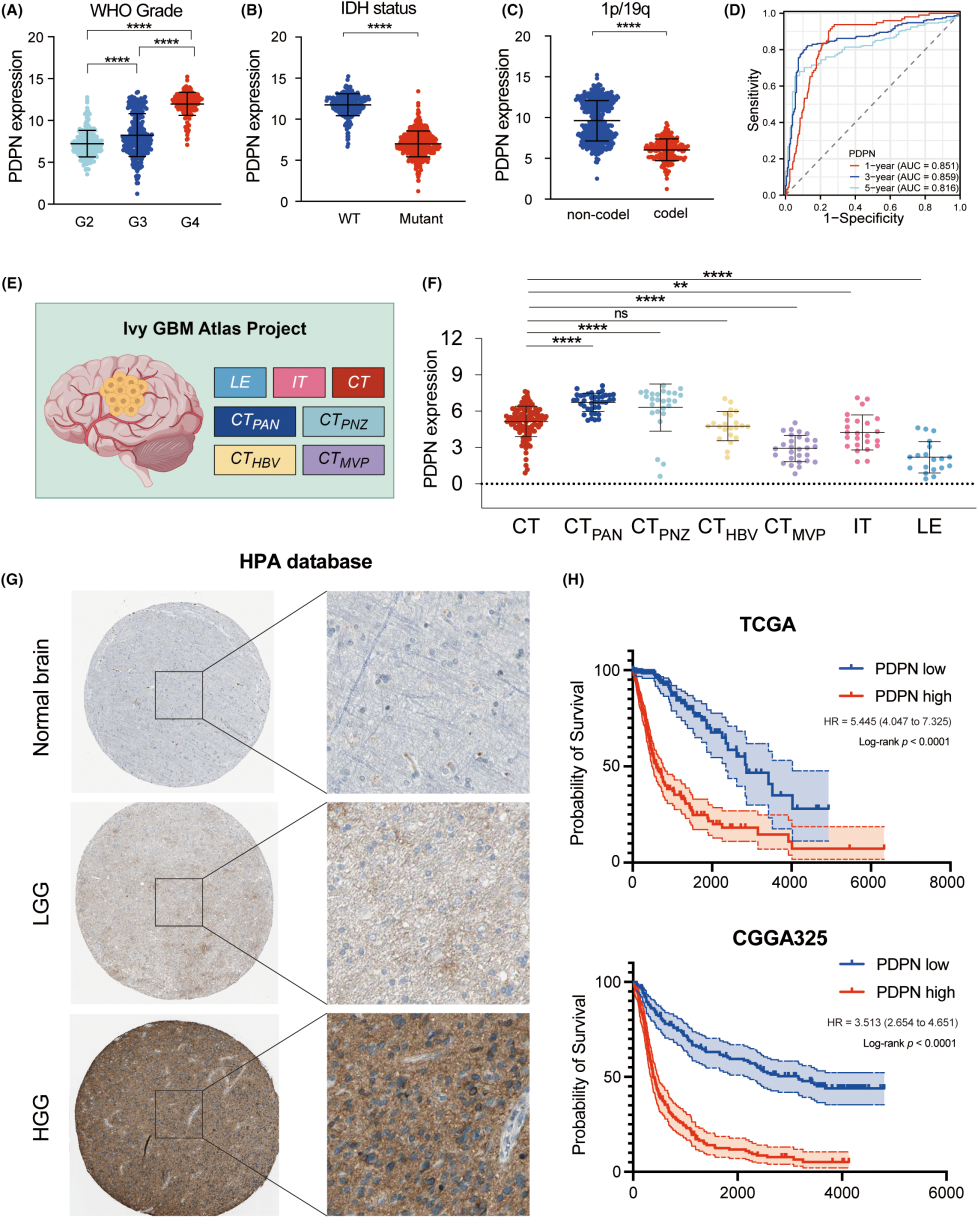
The relationship of PDPN and clinical parameters in gliomas in TCGA and CGGA. (A–C) PDPN expression in groups based on WHO grades (A), IDH states (B), and 1p/19q co‐del states (C). (D) The time‐dependent ROC curves for PDPN. (E) Anatomic structures of GBM tumor based on Ivy GAP database. (F) PDPN expression in the regions of Ivy GAP database. (G) Immunohistochemistry images of normal brain, LGG, and HGG from HPA database. (H) Survival analyses of PDPN‐based groups in TCGA and CGGA325. **p < 0.01, ****p < 0.0001.

The above findings revealed that PDPN might be a potential key factor influencing glioma immune microenvironment. We further explored the expression pattern of PDPN in patients with gliomas and its relationship with prognosis in TCGA. The RNA sequencing data indicated a significant upregulation of PDPN expression with increasing WHO grades (****p < 0.001, Figure 4A). The IDH mutation and 1p/19q co‐deletion in gliomas are recognized as defining molecular markers, indicating improved prognoses and treatment responsiveness. PDPN expression was considerably higher in IDH wildtype and 1p/19q non‐codeletion patients in the TCGA datasets (Figure 4B,C). Furthermore, according to the ROC curve (AUC values = 0.936, 0.978, and 0.896, respectively; Figure S5A–C), PDPN expression might be a robust predictor for WHO grade, IDH mutation and 1p/19q co‐deletion state in gliomas in TCGA dataset. Next, time‐dependent ROC analyses were conducted to evaluate the predictive significance of PDPN in TCGA. The AUC values for 1‐, 3‐ and 5‐year survival of glioma patients were 0.851, 0.859, and 0.816, respectively (Figure 4D). PDPN expression in various anatomical regions of tumors was investigated based on Ivy GAP database. Figure 4E shows the anatomic structures of GBM tumor. The results indicated that PDPN expression in cellular tumor (CT) was significantly higher than infiltrating tumor (IT) and leading edge (LE) (*p* = 0.0012, *p* < 0.0001, respectively). Among the particular structural features of CT region, PDPN expression was relatively high in pseudopalisading cells around necrosis (PAN) and perinecrotic zone (PNZ), and relatively low in hyperplastic blood vessels (HBV) and microvascular proliferation (MVP) (Figure 4F). IHC staining from HPA database suggested higher protein levels in HGG compared with normal brain tissue and LGG (Figure 4G). Lastly, survival analyses indicated a clear association between high PDPN expression and poorer OS in patients from both TCGA and CGGA datasets (Figure 4H, Figure S5D).

### 3.5 Correlations of PDPN expression with macrophages in glioma tissue

**FIGURE 5:**
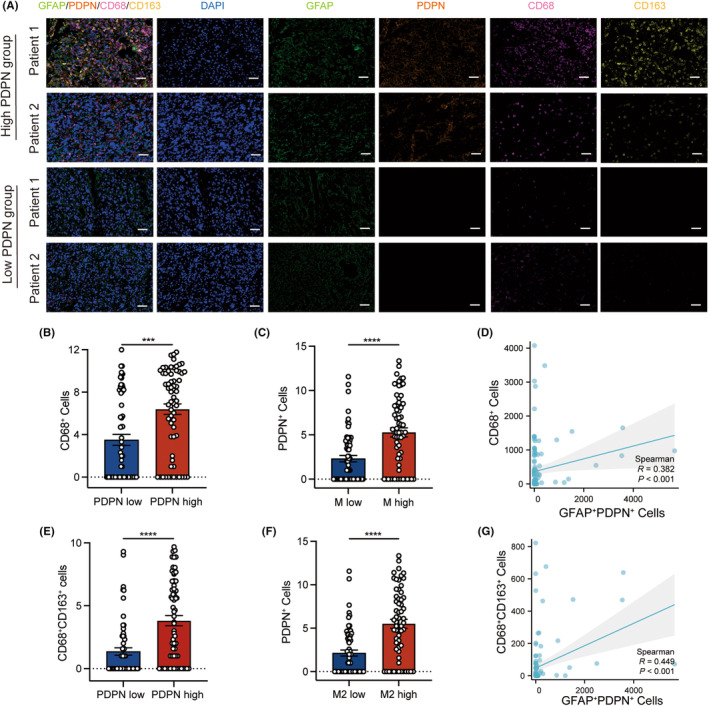
Multiplexed fluorescence immunohistochemistry of PDPN, GFAP, CD68, CD163, and DAPI. (A) MF‐IHC staining of PDPN (orange), GFAP (green), CD68 (pink), CD163 (yellow), and DAPI (blue) in glioma tissue microarray, scale bar 100 μm. (B, E) The number of CD68^+^ (B) and CD68^+^CD163^+^ (E) cells in the PDPN low and high groups. (C) The number of PDPN^+^ cells in the macrophage low and high groups. (F) The number of PDPN^+^ cells in the M2 macrophage low and high groups. The vertical axes of (B, C, E, and F) have been transformed using log_2_(*n* + 1) for better visualization. The cutoff value of (B, C, E, and F) employed the median. (D, G) Linear regression was performed to analyze correlations between cells positive with different proteins in glioma. ***p < 0.001, ****p < 0.0001.

To verify the correlation of PDPN expression and macrophage phenotype, multiplex immunohistochemistry (mIHC) of PDPN, GFAP, CD68, and CD163 was performed with tissue microarray (TMA) containing 3 normal brain tissues and 122 glioma samples. Four representative images, two from high PDPN expression patients and two from low PDPN patients are shown in Figure 5A. High levels of CD68 and CD163 expression, which are M2‐type macrophage markers, were detected in patients with high PDPN expression. However, significantly fewer CD68 and CD163 positive cells were observed in patients with low PDPN expression (Figure 5A). Statistical analysis showed that both CD68^+^ and CD68^+^CD163^+^ cells in the samples of PDPN high group were significantly higher than those in PDPN low group (Figure 5B,E). Besides, more PDPN^+^ cells were observed in samples with higher macrophages or M2‐type macrophages infiltration (Figure 5C,F). Linear regression analysis revealed that the number of CD68‐positive cells was significantly positively correlated with the number of GFAP^+^PDPN^+^ cells in glioma, with CD68^+^CD163^+^ cells showing a similar association with GFAP^+^PDPN^+^ cells (Figure 5D,G).

### 3.6 Tumor‐derived PDPN‐containing EVs mediate M2‐like polarization of macrophages

**FIGURE 6:**
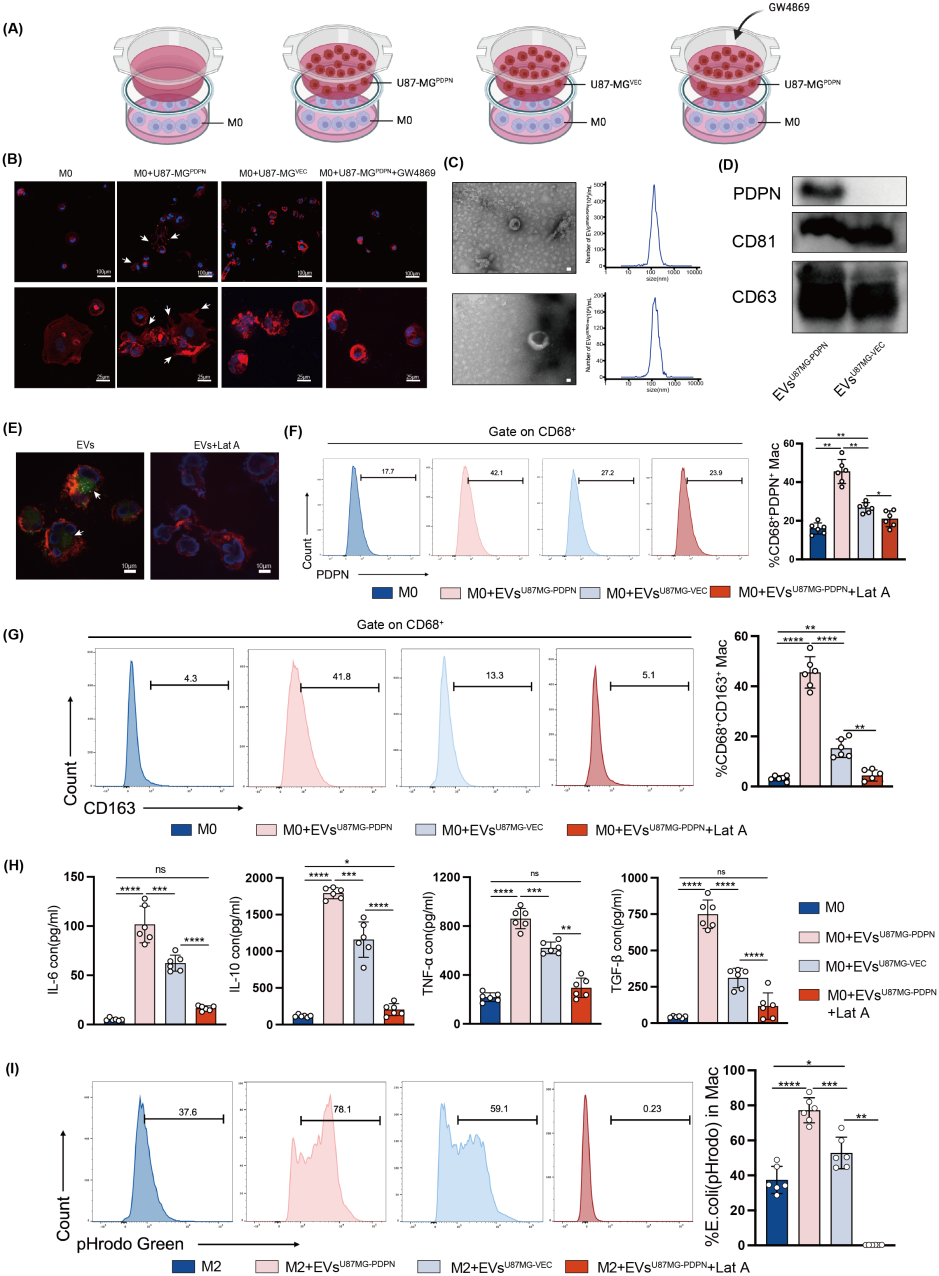
PDPN in GBM cell‐derived exosomes promotes macrophages toward an immunosuppressive phenotype. (A) Coculture model of GBM cells with THP‐1‐derived M0 macrophages. (B) Morphological observation of macrophages after coculture for 48 h, scale bars in (B) up indicate 100 μm, and scale bars in (B) bottom indicate 25μm. (C, D) Characterization of exosomes isolated from U87‐MG^PDPN^ and U87‐MG^VEC^: representative TEM images, nanoparticle tracking analysis (NTA), and expression of exosome marker proteins validated by western blot. (E) PKH67‐label exosomes (green) were phagocytosed by macrophages after coculture for 1 h, scale bar 10μm. (F, G) The percentage of PDPN^+^ macrophages and CD163^+^ macrophages after macrophages cocultured with EVs. (H) The concentrations of immunosuppressive cytokines released by macrophages treated with EVs. (I) The phagocytosis ability of THP1‐derived M2 treated with EVs was examined by PHrodo Green *E. coli* BioParticles using flow cytometry. *p < 0.05, **p < 0.01, ***p < 0.001, ****p < 0.0001.

In our continued exploration of the PDPN‐macrophage axis in vitro, a stable PDPN‐expressing U87‐MG cell line (U87‐MG^PDPN^) was developed through lentiviral transduction. We established a transwell co‐culture system under different conditions as depicted in Figure 6A, which utilizes a semi‐permeable membrane that allows the cell secretome of GBM cells to pass and affect THP‐1‐derived macrophages. To determine whether exosomes affect macrophage polarization, GW4869, an inhibitor of exosome biogenesis/release was also added conditionally to the system. After 48 h of co‐culture, morphological transformations of macrophages were observed. Macrophages co‐cultured with U87‐MG^PDPN^ showed an elongated shape with abundant cytoplasmic projections on the cellular surface, taking on more M2‐like characteristics. A few morphological similarity was presented in macrophages co‐cultured with U87‐MG^VEC^, while the addition of GW4869 showed no significant morphological changes (Figure 6B), which suggested that tumor‐derived EVs might contribute to these changes. EVs were isolated from the supernatant of U87‐MG^PDPN^ and U87‐MG^vec^ with ultracentrifugation. The bilayer‐enclosed morphology of EVs was confirmed with Transmission electron microscopy and particle size analysis showed an average diameter of 148.3 nm in EVs of U87‐MG^PDPN^ and 151.5 nm in EVs of U87‐MG^VEC^ (Figure 6C). Expression of exosome marker protein, CD63, and CD81, was confirmed by western blot. PDPN expression was significantly high in EVs derived from U87‐MG^PDPN^ cell lines, compared to U87‐MG^VEC^ (Figure 6D). After treating macrophages with pure tumor‐derived EVs (50 μg) for 1 h, fluorescent‐labeled EVs were shown to be phagocytosed by macrophages, while no fluorescence was detected in the group treated with EVs and phagocytosis inhibitor, Lat A (Figure 6E). FACS revealed that PDPN‐positive macrophage significantly increased when treated with EVs derived from U87‐MG^PDPN^ (Figure 6F). The percentage of CD163^+^ macrophages presented a similar pattern, indicating that EVs‐PDPN promote the transformation of macrophages into a M2‐like phenotype (Figure 6G). A subsequent assay of the macrophage supernatant revealed elevated levels of inflammatory cytokines, including IL‐6, IL‐10, TNF‐α, and TGF‐β1, when cocultured with EVs carrying PDPN compared with EVs of U87‐MG^VEC^. This trend was reversed by the blockade of phagocytosis with Lat A (Figure 6H). PHrodo Green *E. coli* BioParticles were used to examine the phagocytosis responses. After coculturing with purified EVs (50 μg) for 24 h, pHrodo dye was given to macrophages 2 h prior to cell harvesting. Flow cytometry showed that the presence of EV^U87MG‐PDPN^ promoted phagocytosis greatly (*p* < 0.0001), whereas phagocytosis inhibitor Lat A (30 μM) suppressed the process (Figure 6I). These results consistently showed that tumor‐derived PDPN‐containing EVs were a major modulator of immunosuppressive M2‐like macrophages.

### 3.7 PDPN‐containing EVs regulate MHC II expression and antigen presentation of macrophage via TPL2/Erk/CIITA pathway

**FIGURE 7:**
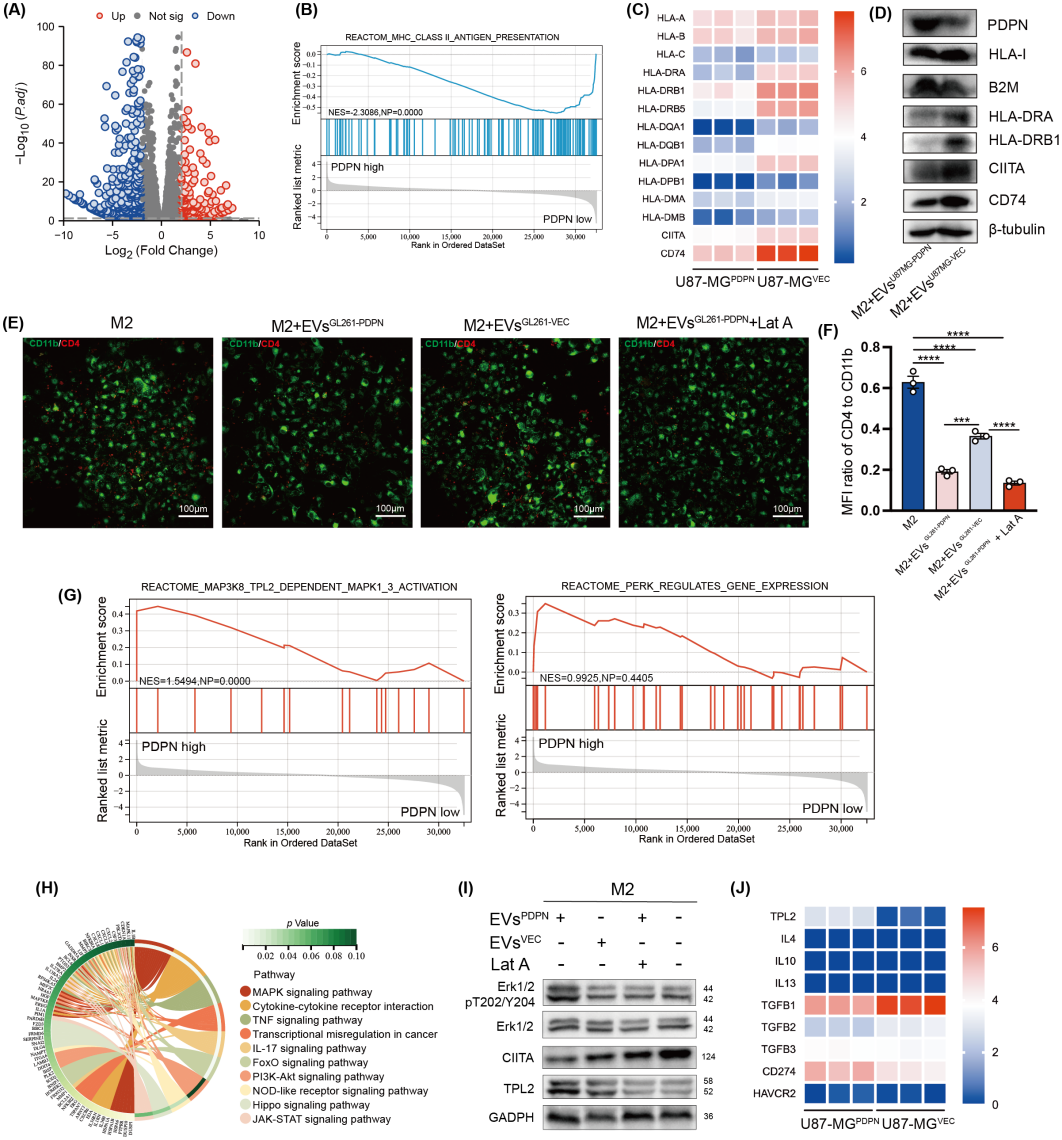
PDPN in EVs promotes immunosuppressive macrophage polarization via TPL2/ERK/CIITA pathway. (A) The upregulated and downregulated DEGs of U87‐MG^PDPN^ and U87‐MG^VEC^ cell lines. (B, G) Enriched pathways were identified by Reactome database based on the ssGSEA analysis. (C, D) MHC gene expression at transcription and protein levels was present. (E) The adherence of OT II T cells with BMDMs was shown in immunofluorescence, scale bar 100μm. (F) The mean Fluorescence Intensity ratio of T cell (CD4: red) and BMDMs‐derived M2 (CD11b: green) was plotted. (H) Chord diagram showing KEGG pathway enrichment of DEGs between U87‐MG^PDPN^ and U87‐MG^VEC^. (I) The activation of TPL2/ERK/CIITA pathway were probed through western blot analyses. (J) Transcription expression of TPL2, M2‐related cytokine and immune checkpoints in U87‐MG^PDPN^ and U87‐MG^VEC^. ***p < 0.001, ****p < 0.0001.

To fully elucidate PDPN‐mediated pathway changes and functional regulation, we compared RNA sequencing data of U87‐MG^PDPN^ and U87‐MG^VEC^ cell lines. Screened with *p* < 0.01; |log_2_FC| ≥ 2, 197 upregulated genes in U87‐MG^PDPN^ compared to U87‐MG^VEC^ and 424 downregulated genes were shown in the volcano plot (Figure 7A). The Gene Set Enrichment Analysis (GSEA) results revealed that the MHC class II antigen presentation pathway was notably suppressed in PDPN‐overexpressing U87‐MG cells, while MHC class I molecules were slightly downregulated (Figure 7B,C). Western blot analysis showed the reduction of MHC II molecules, including HLA‐DRA1, HLA‐DRB1, CD74, and MHC class II transactivator (CIITA), a master regulator of MHC class II gene expression, in M2 macrophage treated with EVs^U87MG‐PDPN^ (Figure 7D). Given the pivotal role of MHC class II molecules in presenting antigens to CD4^+^ T cells, we further investigated whether PDPN compromises the antigen presentation ability of M2 and impairs the activation of CD4^+^ T cells. Bone marrow‐derived macrophages (BMDMs) were isolated from BALB/C mice. Utilizing OT‐II mice, engineered to possess TCR specificity for a chicken ovalbumin peptide, we pre‐treated BMDMs‐derived M2 with EVs isolated from either GL261^PDPN^ or GL261^VEC^ for 48 h. After pulsing with ovalbumin for another 24 h, these BMDMs‐derived M2 were then co‐cultured with OT‐II cells for 1 h. In compliance with decreased MHC II expression, immunofluorescence demonstrated diminished adherence of CD4^+^ T cells to M2 treated with EVs^GL261‐PDPN^ compared with M2 control group and group co‐cultured with EVs^GL261‐VEC^. The group with Lat A maximally blocked the antigen presentation process, resulting in the least amount of T‐cell adhesion (Figure 7E). The mean Fluorescence Intensity ratio of T cell (CD4: red) and BMDMs‐derived M2 (CD11b: green) was shown in Figure 7F. These results suggested that tumor‐derived PDPN‐containing EVs may downgrade MHC II expression and impair the ability of antigen presentation in M2‐like macrophages. To explore the related pathway changes, GSEA analyses were conducted. As shown in Figure 7G, MAP3K8 (TPL2)‐dependent MAPK1/3 activation and Erk phosphorylation were promoted when PDPN was overexpressed. KEGG enrichment analysis consistently suggested the activation of the MAPK pathway (Figure 7H). The regulatory axis of PDPN‐containing EVs, ERK1/2 pathway, and CIITA was investigated.

**FIGURE 8:**
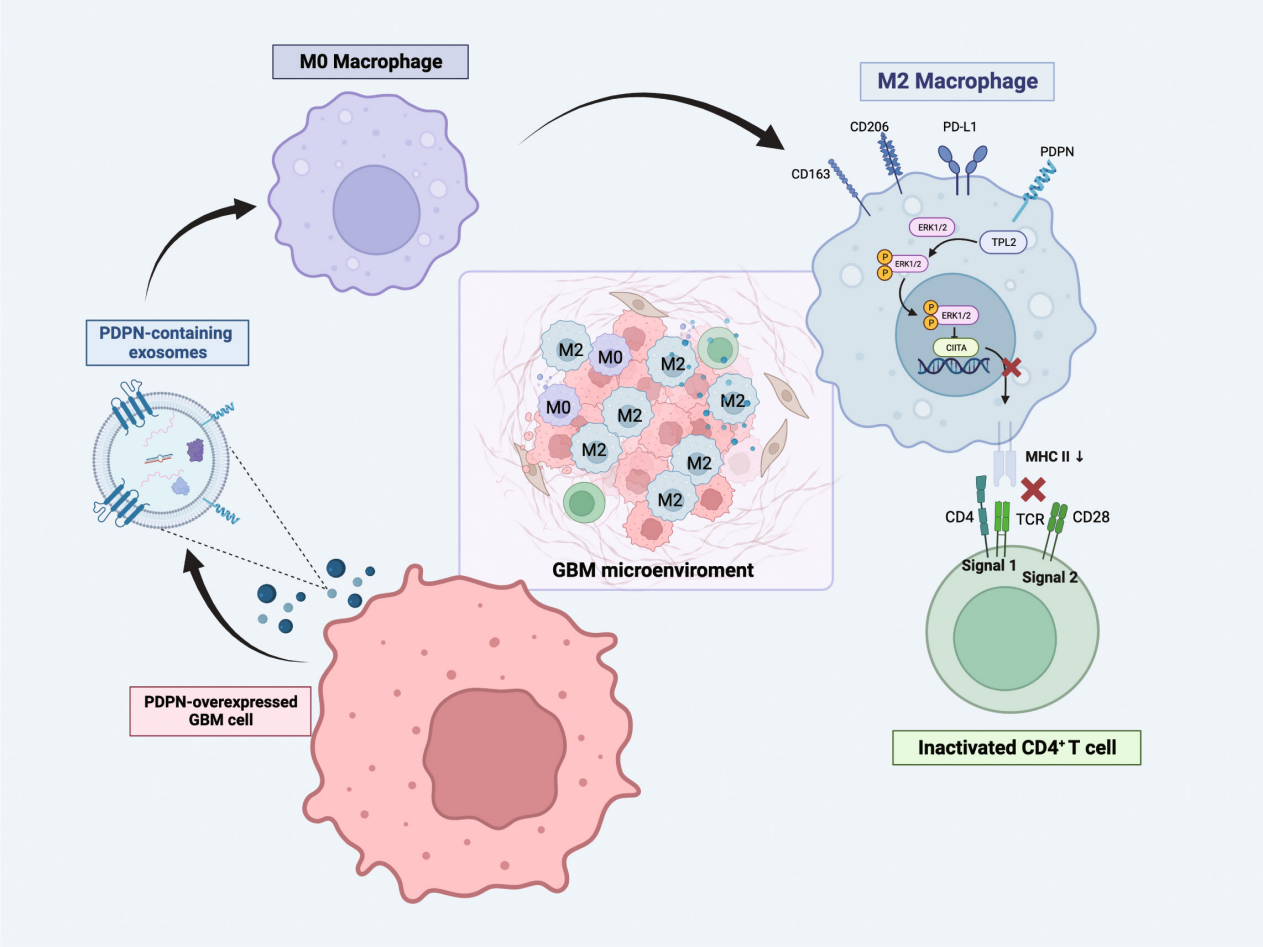
The schematic diagram illustrates the mechanism of PDPN‐mediated macrophage immunosuppressive polarization. PDPN‐overexpressed GBM cells secrete PDPN‐containing EVs, followed by phagocytosis by unpolarized M0 macrophages, which induce immunosuppressive polarization of macrophages, manifested by the release of immunosuppressive cytokines, ERK phosphorylation activation, diminished MHC II expression, and incompetence to CD4^+^ T activation.

TPL2 expression and Erk1/2 phosphorylation in M2 macrophage were upregulated by PDPN‐containing EVs, while CIITA was downregulated. However, the effect was reversed by Lat A (Figure 7I). RNA‐sequencing data also showed that, aside from a consistent upregulation of TPL2, there was no significant difference in M2 polarization‐related cytokines expression between U87‐MG^PDPN^ and U87‐MG^VEC^, including IL‐4, IL‐10, IL‐13, and TGF‐β. At the same time, immune checkpoint molecules CD274 exhibited similar trends in the PDPN‐overexpressing group (Figure 7J). Thus, PDPN‐containing EVs influence macrophages to activate the TPL2/Erk/CIITA pathway and downregulate MHC II molecule expression, leading to the transformation of macrophages into immunosuppressive phenotype (Figure 8).

## 4 DISCUSSION

The current discovery of the intracranial lymphatic system has overturned the concept of “immune privilege” of the brain, establishing a new understanding of intracranial infiltration and function of T cells.^41^, ^42^ Despite this notion shift, clinical outcomes from T‐cell‐based immunotherapies have been largely underwhelming, failing to extend patient survival in glioma.^4^, ^5^ These results suggest that the unique intracranial immune microenvironment disturbs cytotoxic function of T cells. In an open‐label, single‐arm Phase II trial (NCT02337686), immune cell infiltration and immune response in 14 patients with GBM before and after treatment with pembrolizumab were evaluated. The results of CyTOF indicated that there was no significant change in the number of both infiltrated macrophages and T cells before and after immune checkpoint therapy. Remarkably, there are a large number of macrophages that exist in the glioma, accounting for 72.6% of the leukocytes.^43^ Although inherently equipped for innate immune responses, encompassing phagocytosis, cytotoxic activity, and activating other immune cells to participate in the anti‐tumor immune responses, the functionality of glioma‐infiltrating macrophages is partially compromised, presenting with an immunosuppressive phenotype.^44^ Moreover, immune stimulators such as interferon or CpG oligodeoxynucleotide failed to induce MHC II expression in tumor‐associated microglia/macrophages the same as with normal brain tissues.^45^ This may partially account for the scant presence of inactive T cells within glioma tissue, despite the existence of a physiological basis for T‐cell access to the intracranial region. Therefore, targeting on M2 macrophage is supposed to reverse the “immune cold” TME into “immune normalization” TME, improving the outcome of glioma patients. One study showed that radiotherapy promoted the accumulation of M‐2‐like macrophages and that a combination of radiotherapy and macrophage inhibition (using CSF‐1R) delayed glioma recurrence.^46^

Increasing evidence indicates distinct immune cell compositions within various GBM subtypes.^47^, ^48^ The correlation between macrophage infiltration and the molecular heterogeneity of gliomas was established by performing immune analyses on 100 samples from 48 glioma patients. Compared to IDH mutant gliomas that exhibit abundant microglia, there's a higher presence of bone marrow‐derived macrophages (BMDMs) in IDH wild‐type gliomas.^49^ These results suggest that the immunophenotype of intracranial macrophages is strongly influenced by the primary tumor subtype. Therefore, we focused on exploring how macrophages develop immunosuppressive phenotypes in response to specific molecular types of gliomas. The objective is to identify pivotal targets that could yield promising therapeutic strategies.

As a transmembrane protein extensively expressed in stromal cells and tumor cells, PDPN has been documented to modulate platelet activation and tumor‐associated thrombosis. A recent study in melanoma revealed that PDPN functions as an immunosuppressive molecule in T cells. CyTOF analysis suggested that PDPN co‐expressed with immune checkpoints PD‐1, Tim‐3, Lag‐3, and TIGIT on the surface of T cells, and was regulated by the common transcription factors Prdm1 and c‐Maf.^50^ In various brain tumors, such as ependymal tumors, astrocytic tumors, and hemangioblastomas, PDPN overexpression has been observed.^51^ The role of PDPN in immune regulation was gradually discovered. Overexpressed PDPN is reported to be associated with the immunosuppressive tumor microenvironment in GBM, characterized by the interplay between PDPN and M2 macrophage or neutrophil degranulation.^22^ In this study, we found that the membrane protein PDPN, which is highly expressed in GBM cells, is transmitted to macrophages via exosome encapsulation and induces immunosuppressive polarization of macrophages.

A recent study of single‐cell spatial immune landscapes showed significant enrichment of macrophages in the perivascular region,^52^ a distribution not in the tumor core, which has some implications for the way GBM cells interact with macrophages. Long‐distance communication caused by spatial distribution characteristics may be one of the important interaction modes. Recent studies have shown that exosomes play a critical role in the interactions of tumors with macrophages.^10^, ^53^ Specifically, glioma‐derived exosomes have been implicated with immunosuppressive polarization of macrophages via circNEIL3 delivery and stabilization of IGF2BP3.^10^ In addition, studies have shown that tumor‐derived exosomes coated with miR‐3591‐3p induce M2 polarization of macrophages and promote the malignant progression of glioma.^11^ Conversely, tumor‐associated macrophage‐derived exosomal LINC01232 induces immune escape in glioma by downregulating surface MHC I expression.^54^ Therapeutically, targeting PDPN with CAR‐T cells and antibodies has been explored in preclinical research. For example, combination therapy of cancer‐specific anti‐PDPN CAR‐T cells with oncolytic herpes virus inhibited tumor growth and improved survival in GBM.^55^ NZ‐1 antibody and its derivatives can decrease tumor load in xenograft models of glioma.^56^

In conclusion, our research demonstrated the critical role of PDPN in GBM cells in inducing M2 macrophage polarization in an exosome‐dependent manner, contributing to the immunosuppressive milieu characteristic of GBM TME.

## AUTHOR CONTRIBUTIONS

Conceptualization, Ying Shi and Chuan Xu; Data curation, Mengwan Wu and Yuyang Liu; Formal analysis, Mengwan Wu; Funding acquisition, Ying Shi and Chuan Xu; Methodology, Yuyang Liu; Resources, Chuan Xu; Supervision, Chuan Xu and Ying Shi; Validation, Mengwan Wu; Visualization, Mengwan Wu and Yuyang Liu; Writing‐original draft, Mengwan Wu; Writing‐review & editing, Ying Shi, Hongxiang Huang and Jing Shi.

## FUNDING INFORMATION

This study was supported by the National Key Research and Development Program of China (no. 2023YFC3402100) to CX; the National Natural Science Foundation of China (no. 92259102) to CX; Medico‐Engineering Cooperation Funds from University of Electronic Science and Technology of China (no. ZYGX2021YGCX004) to CX; Funds from Sichuan Academy of Medical Sciences, Sichuan Provincial People's Hospital (no. 2022X045) to CX; the National Natural Science Foundation of China (no. 82203539) to YS; Medico‐Engineering Cooperation Funds from University of Electronic Science and Technology of China (No. ZYGX2021YGCX018) to YS.

## CONFLICT OF INTEREST STATEMENT

The authors declare no conflicts of interest.

## Supporting information


Figure S1

Figure S2

Figure S3

Figure S4

Figure S5

## Data Availability

The sequencing data that support the findings of this study have been deposited into CNGB Sequence Archive (CNSA)^57^ of China National GeneBank DataBase (CNGBdb)^58^ with accession number CNP0005041. Additional data are available from authors upon request.
