# Supplementary figures and images for: Exosome‐transmitted podoplanin promotes tumor‐associated macrophage‐mediated immune tolerance in glioblastoma
Source: CNS Neurosci Ther. 2024 Mar 12;30(3):e14643. doi: 10.1111/cns.14643 (PMC10929222; doi:10.1111/cns.14643)

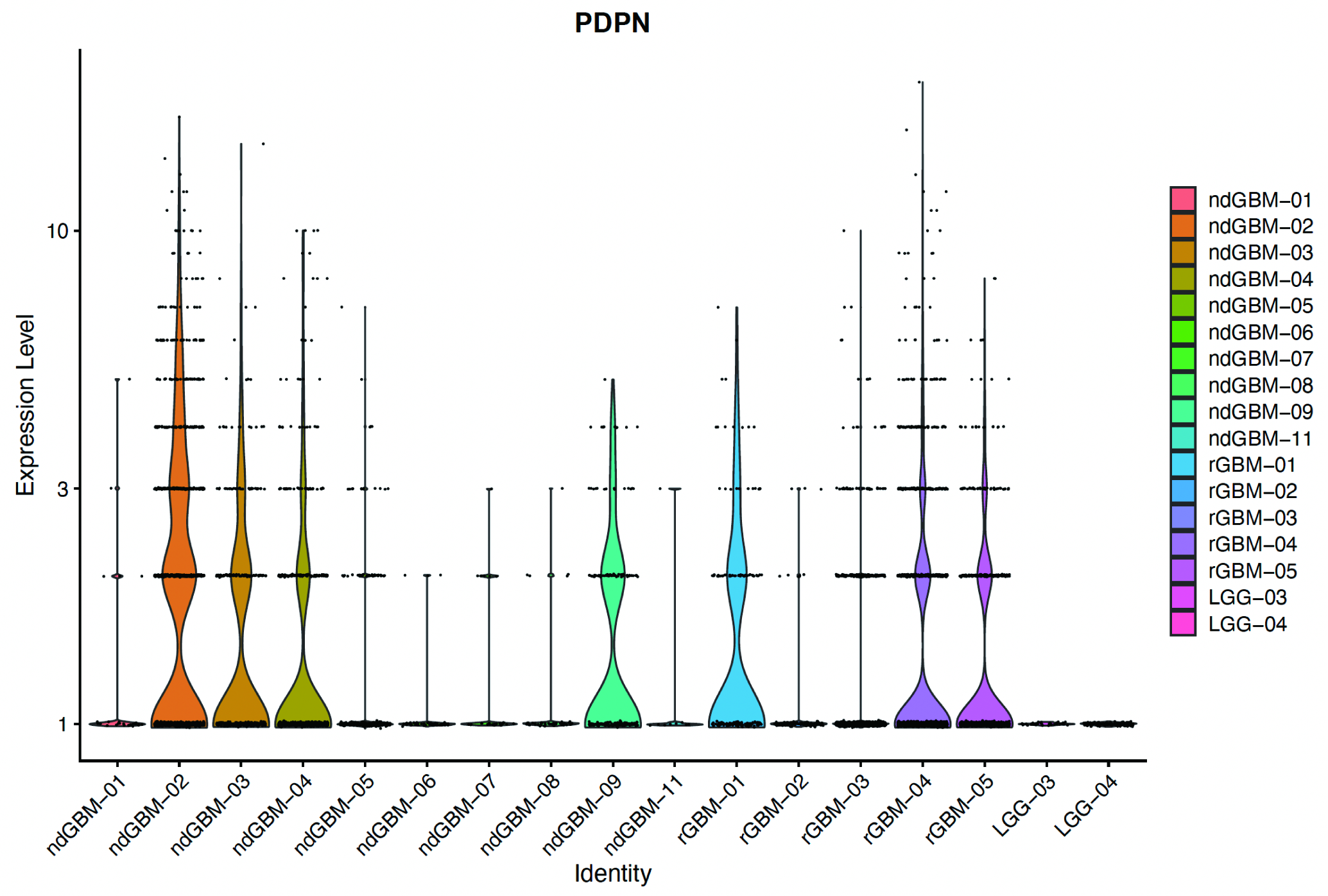

Supplement: Supplementary file 1 — Figure S1 [file CNS-30-e14643-s003.tif]

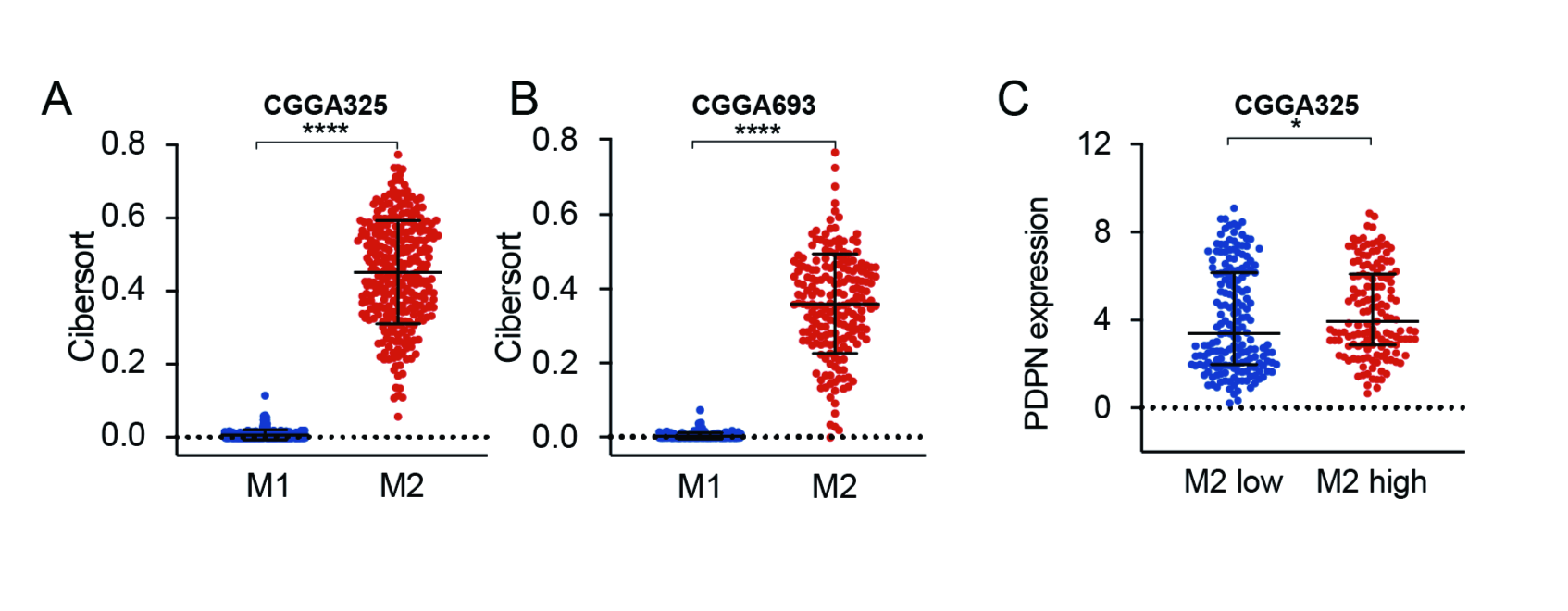

Supplement: Supplementary file 2 — Figure S2 [file CNS-30-e14643-s004.tif]

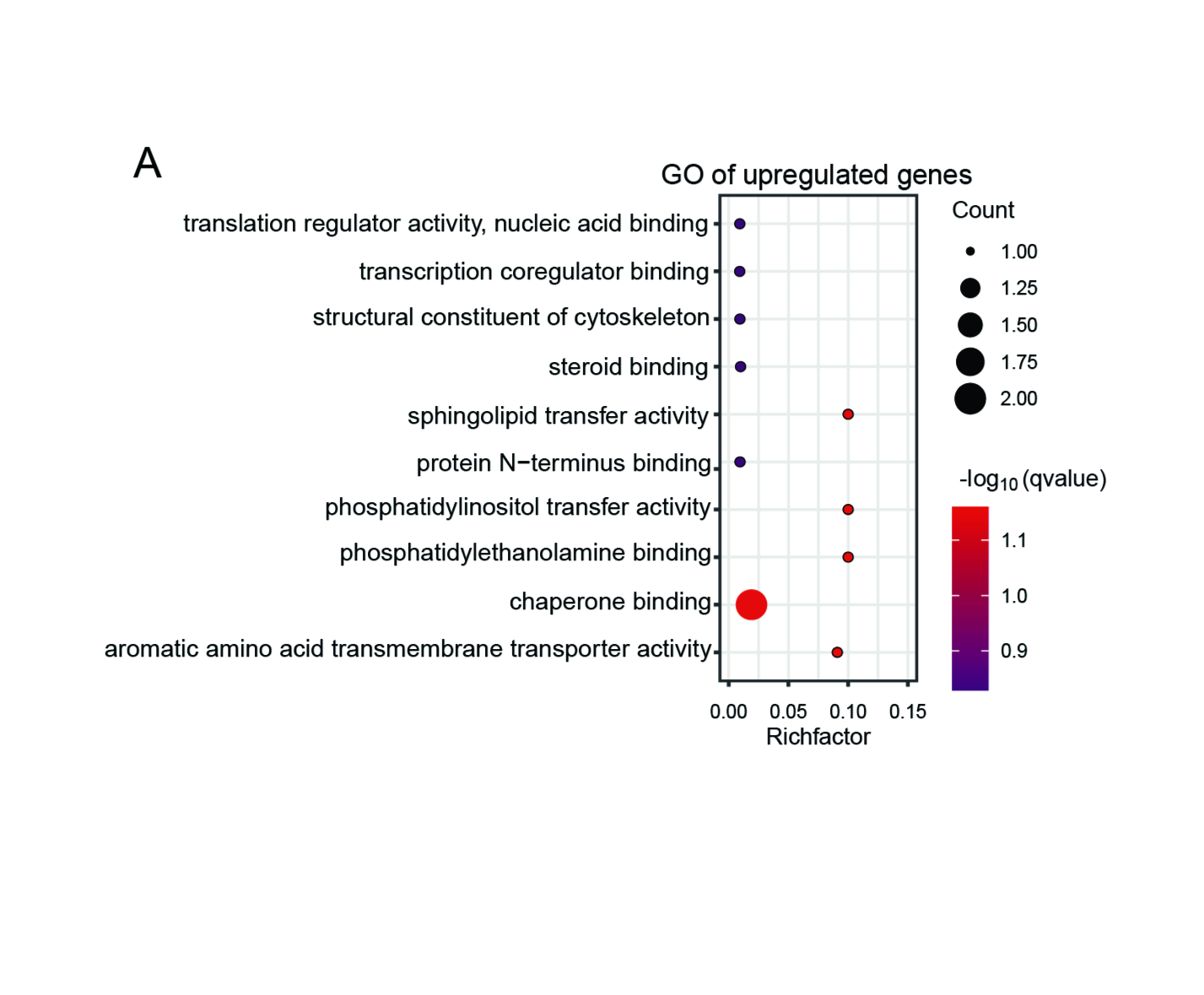

Supplement: Supplementary file 3 — Figure S3 [file CNS-30-e14643-s002.tif]

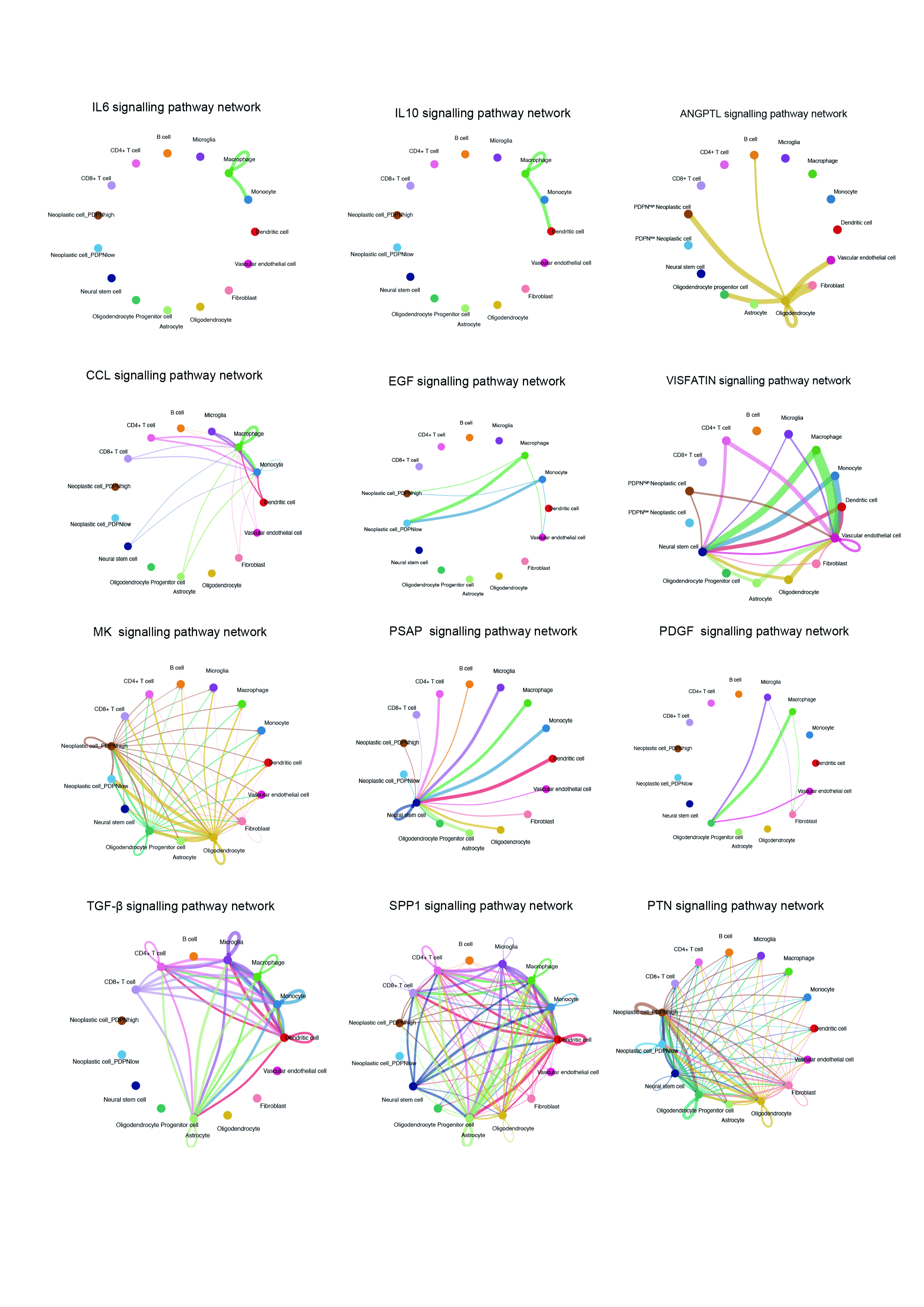

Supplement: Supplementary file 4 — Figure S4 [file CNS-30-e14643-s005.tif]

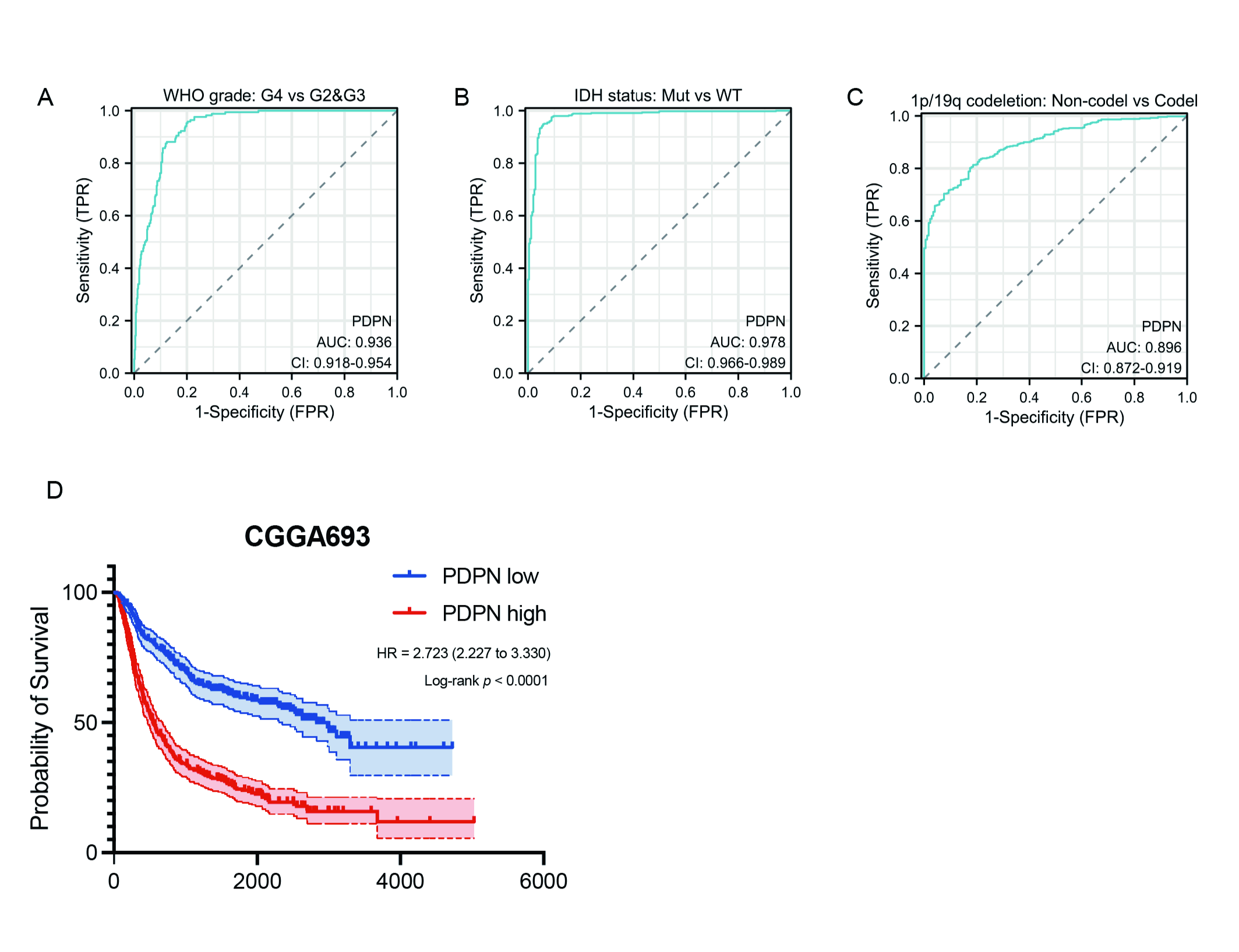

Supplement: Supplementary file 5 — Figure S5 [file CNS-30-e14643-s001.tif]
